# Deconvolution of Red Blood Cells Thermal Fluid Biopsy Following Systematic Cyclophosphamide or Cilostazol Drug Therapies

**DOI:** 10.3390/biology15100792

**Published:** 2026-05-15

**Authors:** Andrea Ferencz, Dénes Lőrinczy

**Affiliations:** 1 Department of Surgery, Budapest Péterfy Sándor Street Hospital and Specialist Clinic, Péterfy Sándor Str. 8-20, H1076 Budapest, Hungary; andrea.ferencz@gmail.com; 2 Department of Biophysics, Medical School, University of Pécs, Szigeti Str. 12, H7624 Pécs, Hungary

**Keywords:** thermal liquid biopsy, red blood cells, peripheral artery disease, cyclophosphamide, cilostazol, hemoglobin, Differential Scanning Calorimetry, deconvolution

## Abstract

Beyond gas transport, red blood cells (RBCs) have emerging roles regarding innate immunity, regulating blood flow, and participating in nutrient transport, which can be engineered as drug delivery systems since they contribute to maintaining water homeostasis. Following extensive thermoanalytical studies of human blood plasma, our working group investigated the properties of RBCs, examining their role in healthy and in different disease states by using differential scanning calorimetry (DSC) and the deconvolution of the resulting thermal curve. In the first study, guinea pigs were treated with intraperitoneal chemotherapy. Cyclophosphamide treatment showed a dose-dependent difference between the thermal parameters of control and treated samples, indicating that DSC can be used in this area. Following deconvolution of the DSC studies, the changes can be attributed to the damaged compounds. In the second part of our study, a method for the thermal analysis and deconvolution of RBCs in patients with lower limb ischemia during a three-month cilostazol treatment was developed. The control DSC curve showed 5–6 distinct thermal domains, and in contrast to other drug treatments, this remained stable throughout the entire study period. No effects of stiffness or compact were caused by the anticancer drug cyclophosphamide were observed in the structure of RBCs. These preliminary results highlight the uniqueness of thermodynamic studies of RBCs and provide a fingerprint-like identification of a given individual or disease state.

## Simple Summary

Red blood cells (RBCs), which give blood its hue, play a fundamental role in regulating blood flow, transporting respiratory gases, and thus the functionality of the complex animal and human body. Our research group used differential scanning calorimetry (DSC) to study the properties of RBCs to analyze their role prior to and following the administration of two drugs that have opposing effects upon the RBCs structure and function. During cyclophosphamide chemotherapy, a dose-dependent difference was observed between the thermal parameters of the control and treated samples, indicating the negative effects of chemotherapy on the isolated RBC fraction. In the second part, we performed thermal analysis of RBCs in patients with lower limb ischemia undergoing three months of cilostazol treatment. The control DSC curve exhibited 5–6 distinct thermal bands, and unlike with other drug treatments, this remained stable throughout the entire study period. All of this indicates one of the drug’s key effects is the preservation of the structural stability of RSCs, which also ensures its long-term efficacy in the body. These results highlight that thermodynamic analysis of RBCs can provide unique identifiers for detecting the effects of therapeutic agents in the study of a given individual or disease, making erythrocytes a crucial blood component for such investigations.

## 1. Introduction

It is a well-known fact that red blood cells (RBCs) play an essential role in oxygen transport and in maintaining tissue oxygen balance and homeostasis. Each erythrocyte contains millions of hemoglobins (Hb) molecules, which give blood its red hue. The Hb protein consists of four units, each of which is composed of heme, an iron-containing subunit, and a globin subunit. Its primary function is to transport oxygen, but it also binds other gas molecules (e.g., carbon dioxide, carbon monoxide) [1].

According to the latest research, RBCs possess more than this traditionally recognized function. Recent studies highlight that understanding the effects of systemic stress factors on circulating RBCs can provide valuable insights into the broader context of health and disease. Clarifying the molecular complexities of these relationships will not only advance our understanding of fundamental biological processes but will also pave the way for the development of targeted therapeutic interventions which mitigate the harmful effects of oxidative stress on RBCs and, more broadly, on overall health [2,3].

Recently, differential scanning calorimetry (DSC) is an increasingly used measurement method for diagnosing various diseases (structural transition in RBC [1], identification of cancer in plasma and serum [2,3,4,5,6,7]). Its fidelity is around 80%, of which the 60–80 °C range [8] is currently used in the method referred to as thermal liquid biopsy (TLB) [9]), implemented when examining the effects of drugs used in their treatment (cyclophosphamide in chemotherapy [10], Parkinson’s disease [11], neurodegenerative pathologies and aging [12], cancers [13], and cilostazol in PAD patients [14]). The advantage of the method is it is fast, requires a small sample size with routine blood sampling, is inexpensive, and is the only method which directly measures the change in heat capacity. If any structural change occurs in a molecular system (due to disease, drug treatment, etc.), it causes a change in heat capacity, which is measured by the system as a function of temperature. Our present study utilized our previous measurement data to present two cases from our investigations into RBCs. Cyclophosphamide was administered to animal samples, while cilostazol was administered to human subjects.

During life processes, the substances necessary for the functioning of cells/organs reach their points of use through blood circulation. These interact with the various components of the blood, the effects of which can be traced through the two examples highlighted below.

According to the Food and Drug Administration (FDA), cyclophosphamide is mainly indicated for use as a chemotherapeutic agent in the treatment of different malignant lymphomas, breast cancers, and ovarian adenocarcinomas in advanced stages. Moreover, it has also been applied as an immunosuppressant to prevent transplant rejection and graft-vs-host complications [15]. Cyclophosphamide is an inactive prodrug that exerts its antitumor effects by metabolizing 75% of it by cytochrome P-450 isoforms (CYP3A4, CYP2C19, CYP1A2) liver enzymes, which leads to the formation of phosphoramide mustard (PAM).

This active metabolite form is transported via the bloodstream to reach tumor cells, where they are taken up by organic anion transport peptides and other transporters. PAM connects with irreversible cross-links at the N-7 position of guanine in the DNA double helix, which ultimately leads to programmed cell death [16]. According to our pharmacokinetic knowledge, PAM (half-life 3–11 h) as an alkylating agent has high bioavailability, low protein binding, and significant inter-patient variability in metabolism. Patients administered cyclophosphamide require regular monitoring of RBC counts, including hemoglobin and reticulocytes, particularly in high-dose treatments. A main cyclophosphamide metabolite, PAM binds to hemoglobin to form PAM-Hb, a stable biomarker used to monitor drug exposure [17].

Cilostazol is an antiplatelet and vasodilator agent used to treat intermittent claudication; it inhibits the activity of type 3 phosphodiesterase by increasing intracellular cyclic adenosine monophosphate (cAMP) levels. Additionally, cilostazol exerts a range of pleiotropic effects, including anti-apoptotic, anti-inflammatory, antioxidant, and cardioprotective actions. It increases high-density lipoprotein cholesterol levels and reduces triglycerides; consequently, its effects on atherosclerosis and its potential applications in other conditions resulting from atherosclerosis, such as secondary ischemic cerebral infarction and hemorrhagic stroke, are currently the subject of intensive research [18].

In this review, we evaluate our previous measurements according to new aspects since we focus on measurement data applying cyclophosphamide on animal experiments and cilostazol on human examinations. Specifically, when the deconvolution of DSC curves was examined using the Sanchez-Ruiz activation energy method, it revealed consistent changes in the thermal denaturation of RBCs.

## 2. Materials and Methods

### 2.1. Animals

Cavia porcellus from a local colony were used for our experiments (*n* = 60), since Guinea pigs have been used as a “human model” in medical research for decades, and many of their physiological characteristics are like those of humans. They are particularly important in immunological research, which is why we tested the effect of cyclophosphamide on guinea pigs. Animal housing, care, and application of experimental procedures were in accordance with institutional guidelines under approved protocols. Animals were fed and watered ad libitum under light/dark cycles of 12/12 h. The experimental animals were treated via intraperitoneal injection—considered usual in the case of drug administration for pets [19]—for 12 different dosing schemes (*n* = 60, *n* = 5/group, see [9]). The dose of cyclophosphamide was normalized to the guinea pigs’ mass according to the human protocols (150 mg kg^−1^ b.m.). Injection was administrated 1–6 times and included several days of breaks [9].

Recently, we focused on the results of the next samples: control and 1-, 3-, and 5-injection treatments. Untreated guinea pigs were used as controls (*n* = 5). All procedures were performed in accordance with the ethical guidelines approved by the University of Pécs (permission number: BA02/2000-4/2012).

### 2.2. Patient Population

Five males and five females, with a median age of 58.6 years, were involved in this study having intermittent claudication, as well as age-matched healthy control individuals (*n* = 5) [14]. Exclusion criteria were aged under 40 and over 75 years, walking distance limitation under 400 m, and lack of pedal pulses. Individuals who had severe ischemic heart disease as well as chronic kidney failure or limb-threatening ischemia were also excluded. Due to the small number of patients, we did not make age quartiles. The patients administered 100 mg of cilostazol orally, taken twice daily. Patients attended clinical visits after 2 weeks, 1 month, 2 months, and 3 months. The routine assessment of the drug’s effect was tested by the change in pain-free walking distance [14], in which no other clinical parameters were examined. This study received ethical approval from the Hungarian Medical Research Council (IV/2448-4/2022), and all participants provided written informed consent.

### 2.3. Blood Sample Collection and Preparation

Sample preparation in detail can be found in [9,14]. At the conclusion of the experiments, guinea pigs were over-anesthetized. Peripheral blood samples were collected from all treated and control animals and from drug-receiving and healthy controls. Blood samples were collected into Vacutainer tubes containing EDTA (1.5 mg mL^−1^ of blood) centrifuged at 1600× *g* for 15 min at 4 °C to separate the plasma fraction from red blood cell components. We used the generally proven RBC sample preparation: following centrifugation and separation, the RBCs were washed in PBS buffer and stored in an ice water bath at 4 °C prior to DSC measurement, which was performed within several hours. During the denaturation measurements, the calorimetric enthalpy was normalized to the measured wet RBC mass.

### 2.4. DSC Measurements

The thermal unfolding of the RBC components were monitored using a SETARAM Micro DSC-II calorimeter (Setaram Instrumentation, Caluire-et-Cuire, France). All experiments were conducted between 0 and 100 °C. The heating rate was 0.3 K min^−1^ in all cases. Conventional Hastelloy batch vessels were used during the denaturation experiments with 850 μL of sample volume on average. A reference sample was normal saline (0.9% NaCl). The sample and reference samples were equilibrated with a precision of ±0.1 mg. The repeated scan of denatured sample was used as a baseline reference, which was subtracted from the original DSC curve. We plotted the heat flow (DSCII is a heat flux instrument with hermetically closed vessels) regarding the function of temperature. Calorimetric enthalpy was calculated from the area under the heat flow–temperature curve by using two-point-setting SETARAM peak integration, and it was normalized on the wet mass of the used RBCs.

### 2.5. Deconvolution of DSC Thermal Curves

Thermal data for various components of RBCs are known from previous denaturation studies [1,20,21,22]. Using these *T_m_* values, the average experimental DSC scans were decomposed into the sum of Gaussian curves to assure the sum of these functions would best approximate the areas under the experimental curves, i.e., the value of the calorimetric enthalpy. The area of these putative structural (thermal) domains is proportional to their contribution to total calorimetric enthalpy. The change when compared with controls can be used to infer the effect of the intervention/treatment. In Tables 2–4, we only considered deconvolved data that were above the instrument’s enthalpy determination uncertainty (error, which is ~3–5%) in this case.

### 2.6. Statistical Evaluation

To examine the alteration among data, we performed an unpaired, independent post hoc two-sample *t* test for each sample series from Table 1, using the means and standard deviations of the groups containing five samples to determine the differences between all possible experimental conditions.

## 3. Results

### 3.1. Cyclophosphamide Treatments of Guinea Pigs

**Figure 1:**
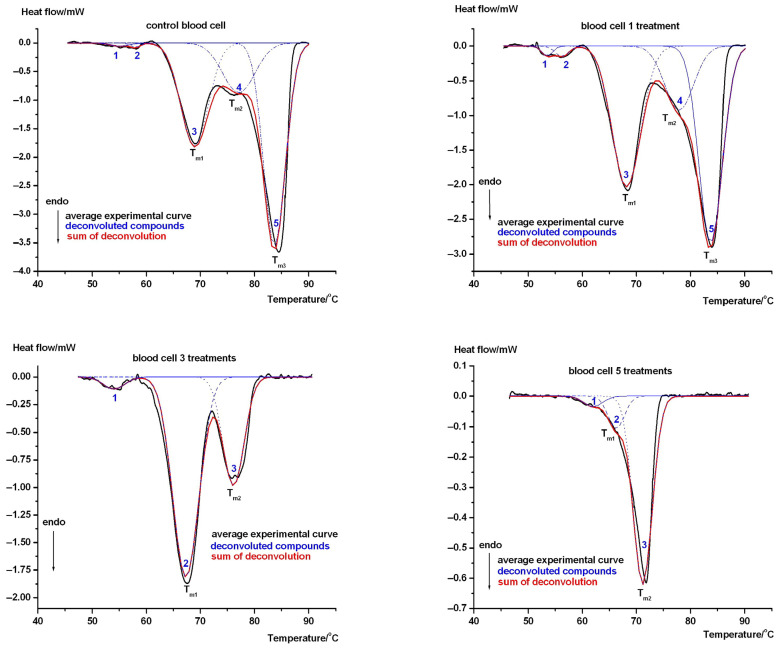
The decomposed denaturation scans of guinea pig RBCs on controls and following 1, 3 and 5 cyclophosphamide treatments (black is average of experiments, blue dashed is deconvolved thermal domains, red is their sum). The numbers indicate the major structural units (so-called thermal domains) that can be identified based on other studies, while T_m1_…T_m3_ are the denaturation temperatures of the domains that provide the largest enthalpy contribution.

In Figure 1, the deconvoluted denaturation scans of the most representative cyclophosphamide treatments of guinea pigs can be seen. The fidelity of the deconvolution is around R~0.95 (the difference between the area of measured denaturation scan and the sum of fittings curve differs by ±5%). According to the published literature, DSC scans can have up to four distinct thermal domains, which include hemoglobin (~70 °C), the membrane-spanning domain of B3 glycoprotein (at ~63 °C), bands 2.1, 4.1, and 4.2 proteins (~57 °C), and spectrin (~55 °C), just like in human samples [1,20,21,22,23]. In our case, we discovered a fifth one (above 80 °C), of which the identification/interpretation requires further investigation.

The effect of chemotherapy treatment on RBCs can also be seen from the temperature dependence of the DSC scans (the course of the curves). Table 2 shows the thermal domains that made the largest contribution to the heat of denaturation; therefore, we focused on the range above 60 °C—marked *T_m_*_1_*-T_m_*_2_*-T_m_*_3_ in Table 2, after cyclophosphamide treatment.

According to the denaturation data from human samples and our animal results, *T_m_*_1_ (see Table 2) can be attributed to Hb contribution. Its enthalpy contribution to the total one increases with each added number of treatments up to three injections. Despite the decreasing trend in *T_m_*_1_, the increased enthalpy is the sign of compacted internal RBC structure due to an increased amount of bounded cyclophosphamide to Hb during treatment.

Following three and five injections, the denaturation peak above 80 °C disappeared (in recent investigations, we could not identify its source). The *T_m_*_1_ (see Table 2) and the total calorimetric enthalpy decreased to a minimum upon conclusion of treatment, indicating structural relaxations due to the chemotherapeutic effect. While the enthalpy contribution of hemoglobin was twice that of control in the case of three injections in *T_m_*_1_, in the case of five treatments, it seems Hb had two different thermal domains. T_m2_ exhibits a strongly bound Hb-cyclophosphamide complex with *T_m_*_2_ = 71.8 °C and due to enthalpy contribution increased threefold when contrasted with the control. It is very likely around 67 °C and with an enthalpy contribution measured at 14%, is very loosely joined to the core. It could represent the increased deposition of the cyclophosphamide in Hb with decreased transport to the target cells.

### 3.2. Cilostazol Treatments of Patients

**Figure 2:**
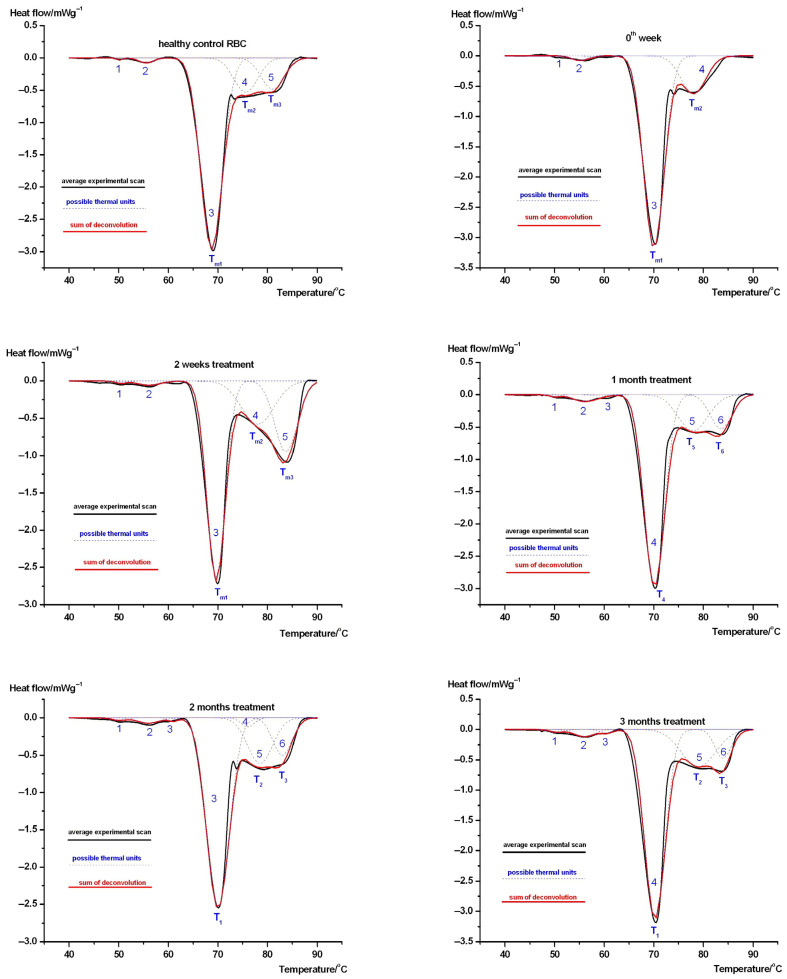
The deconvolution of average denaturation scans of cilostazol-treated female patient’s RBCs (black is average of experiments, blue dashed lines are deconvolved thermal domains, and red is their sum). Symbols mean the same as in Figure 1.

**Figure 3:**
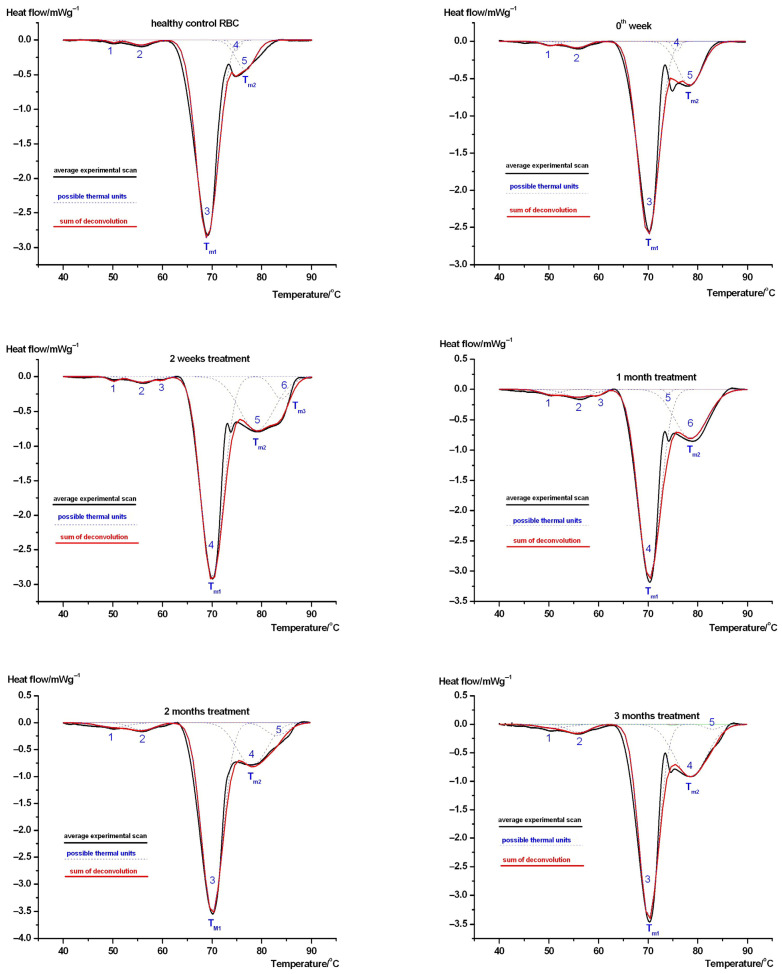
The deconvolution of average denaturation scans of cilostazol-treated male patient’s RBCs (black is the average scan of experiments, blue dashed lines are deconvolved thermal domains, and red is their sum). Symbols mean the same as in Figure 1.

Figure 2 and Figure 3 represent our results in the case of cilostazol-treated female (Figure 2) and male (Figure 3) patients. Zero weeks presents initiation of the treatment. In these cases, we also focused on the larger effects in the case of marking the denaturation temperatures in the tables.

As in the case of animal experiments in human beings, we have also found three significant (above the experimental error) contributions to calorimetric enthalpy in the case of female patients (see Table 3).

The unidentified T_m3_ effect contributed to the calorimetric enthalpy with ~10% (error~5%) with exception to the initiation of the treatment (0th week) and increased to ~23% following 2 weeks of treatment, which may be the consequence of a “shock” effect due to medical treatment. The Hb very likely exhibits two thermal domains with T_m_s~70 °C and 78 °C. The enthalpy contribution of the lower one is decreasing, referring to healthy and 0th week data (patients exhibited higher values prior to treatment when contrasted with healthy controls), while the higher one gives increasing ΔH_c_ contribution with increased denaturation temperatures. This most likely means during treatment, the bound drug can make a more stable and increasing core with a decreasingly stabilized remaining part of Hb. The total calorimetric enthalpy monitored the treatment in the following way: except for the second week and first month treatments, there was a significant difference between healthy controls and treated patients (*p*~0.02). Among treated patients, there was no significant difference between the 0th week and 3rd; however, a significant difference was found in all other treatment time combinations (*p*~0.02). The T_m1_ denaturation temperature proved to be the least sensitive to treatment, with a significant difference (*p* < 0.001) compared only with healthy control. In the case of T_m2_ denaturation temperature, we observed a significant effect between the 0th week and 3rd month as well as between the month 1 and 2 and month 2 and 3 treatments (*p* < 0.03). In the case of T_m3_, there was no significant difference only between months 1–2 and 1–3; however, there was a significant difference for all other treatment protocols (*p* < 0.004).

In the case of male samples, the scans showed similar results as in the case of females (see Figure 2 and Figure 3). The total calorimetric enthalpy monitored the treatment in the following way (see Table 4): unlike female samples, healthy controls were significantly different in all treated patients and referred to the starting stage of treatment among the patients (*p* < 0.001). Among treated patients, there was no significant difference, only between 1–2 and 1–3 months. The T_m1_ denaturation temperature exhibited a significant difference (*p* < 0.001) when compared only with the healthy control. It was comparable with the female results. In the case of T_m2_ denaturation temperature, we observed only significant alteration between the 0th and 2nd week and between month 1 and 2 and month 2 and 3 treatments (*p* < 0.03). In the case of T_m3_, the enthalpy contribution was in the range of measurement error, of which its interpretation was invalidated.

The effect of cilostazol showed a dependence on sex and thermodynamic parameters (see Table 3 and Table 4). While the denaturation temperatures of healthy controls in both sexes significantly differed from similar values in treated patients at all time points of the study, indicating the effect of the disease on RBCs regardless of sex, the calorimetric enthalpy in males showed a significant difference (*p* < 0.04) when compared with the control at all time points, yet among females only at months 0, 2 and 3. In the treated female samples at *p* < 0.03, the data for 0–1–2 months exhibited a significant difference, while for males it was at *p* < 0.01 except for the range of 1–2–3 months. In the case of treated patients, there was no significant difference in the *T_m_*_1_ parameter between females and males during the study period. In the case of *T_m_*_2_, in the male group, the data for 0–2 weeks and 1–2 and 2–3 months were significantly different regarding *p* < 0.03. In the case of females, the data for 2 weeks to 3 months, 1–2–3 months, and 2–3 months were significantly different regarding *p* < 0.03. In the case of *T_m_*_3_, we experienced a significant difference between 2 and 3 months in females and between 2 weeks and 2 months in males regarding *p* < 0.004. In thermal analysis, the denaturation temperature and calorimetric enthalpy can be related to the mechanical properties of the sample in the study. It is generally accepted that an increase in these parameters during the intervention indicates a more ordered, stiffer structure. In one of our recent works with Type 1 diabetes mellitus from 2025, we had the opportunity to perform DSC and microscopic examination of RBC, in which the microscopic images showed a deformation change confirming the direction of thermal effect in diabetic patients.

## 4. Discussion

Cyclophosphamide is a widely used drug that was first synthesized by Friedman and Seligman in 1954 and is now included in the newest WHO Model List of Essential Medicines for 2025 [24,25]. It has indications for use during treatment of immunological diseases (systemic lupus erythematosus, vasculitis, myositis, and scleroderma), various types of cancers (lymphoma, myeloma, leukemia, breast cancer, ovarian cancer, small cell lung cancer, sarcoma, nephroblastoma, rhabdomyosarcoma), and organ transplant recipients. Both intravenous and oral formulations are administrated in daily practice. While both approaches offer several beneficial effects, they are also associated with numerous short- and long-term side effects (pancytopenia, loss of appetite, vomiting, hair loss, allergic reactions, etc.). Its pharmacokinetics and metabolism exhibit a high degree of variability; therefore, it is quite difficult to predict its therapeutic effects and side effects [9,26,27,28].

In recent decades, numerous studies have been published regarding the use of DSC to examine various components of blood. These studies have demonstrated that thermal analysis is a highly sensitive and informative technique for detecting changes in blood plasma, serum, and RBCs to detect changes caused by various diseases or treatments [11,12,13,14,15,16,17,18,19,20,21,22,23]. If cyclophosphamide binds to RBCs, its metabolites cause cross-linking of RBCs membrane proteins (such as spectrin) and polymerization of proteins in the RBC cytoplasm. These changes contribute to the modification of RBC membrane structure and potential damage, which can alter the stability and flexibility of the RBCs’ membrane. Surprisingly, there is little new scientific data referencing this subject in the published literature [29,30]; however, these changes must occur in the cell membrane, and these changes should be evident when comparing DSC analyses of treated and control RBCs. We had already detected all these features in the averaged DSC scan; however, their “unfolding” using deconvolution of the experimental curves becomes increasingly more visible [26].

Much as in the case regarding human samples, the DSC scan of red blood cells from the control group in our animal experiment on guinea pigs clearly shows five thermal transitions. As in other studies, five distinct components emerged upon deconvolution of the main thermal curves (black line in Figure 1). According to the thermal denaturation and based on the literature data, the following RBC components have been identified: spectrin (~55 °C, 1 in Figure 1 control), band proteins (~57 °C, 2 in Figure 1 control), and the transmembrane domain of the B3 glycoprotein (~63 °C, 3 in Figure 1, shifted to ~68 °C). The main unbound protein of RBCs, the free hemoglobin molecule, appears around ~70 °C (4 in Figure 1, shifted to ~76 °C), while the contribution of the bound Hb complex can be detected above 80 °C (5 in Figure 1, shifted to ~83 °C) [29,30], which is also supported by similar DSC results obtained on other diabetic RBCs, in which microscopic studies showed structural mechanical changes [31].

Following a single dose of cyclophosphamide treatment, distinct changes can be observed during the DSC curves when compared with the control (see Figure 1: one and two peaks). Regarding the contribution of spectrin and bands 2.1, 4.1, and 4.2, proteins are elevated at more than 80% [28], indicating a more stable structure. Spectrin is a crucial cytoskeletal protein forming a flexible, hexagonal meshwork on the inner membrane of RBCs. It enables RBCs to deform while traveling through capillaries and maintains their biconcave shape. Defects in spectrin lead to hereditary hemolytic anemias such as spherocytosis or elliptocytosis. These conformational changes may play a role in the development of RBC dysfunction and anemia. Additionally, the fifth transition component (above 80 °C) can be identified as a possible cyclophosphamide-Hb complex [29,30,31].

The most pronounced effect of cyclophosphamide begins following three or more treatments. Following three injections, only three thermal transitions can be identified: the contribution of spectrin and bands 2.1, 4.1, and 4.2 proteins merged (with T_m_~54 °C), which cannot be separated, similarly to the merging of membrane-spanning domain of B3 glycoprotein and hemoglobin (~67 °C). The contribution of the possible cyclophosphamide binding domain appeared with extremely decreased enthalpy contribution and decreased T_m_, indicating severe chemotherapy damage (see Figure 1). This trend continued following five therapeutic dosages, in which the second and third curves following three treatments completely “merged”. Interestingly, if there are more than three treatments, the cyclophosphamide-Hb complex as a fifth peak no longer appears as a separate component: rather, it “merges” into a total compact structure. Moreover, when compared with the control calorimetric enthalpy, while the first drug treatment increased it, the subsequent three and five treatments significantly reduced the total calorimetric enthalpy regarding these thermal units, indicating a serious harmful effect of chemotherapy treatment.

Current studies indirectly reveal fine details regarding the cellular changes underlying these treatments, which otherwise remain unknown. It is a well-known fact in clinical practice that when there is a decrease in an absolute account of RBCs, systemic cancer treatments are often accompanied by qualitative changes in red blood cell morphology and membrane integrity. According to some studies, in leukemia patients, red blood cells often lose their characteristic “doughnut” shape, and irregularities, increased porosity, and an irregular appearance can be observed on their membrane surface. These morphological changes stem from disturbances in the organization of the red blood cell membrane, which plays a critical role in regulating cell deformability in pathological conditions, including anemias [27,28,29,30,31,32]. Furthermore, deformability, which is the ability of erythrocytes to undergo reversible shape changes in response to mechanical forces, —is a critical determinant regarding microvascular perfusion and oxygen supply. As a result of antitumor therapy, RBCs undergo qualitative changes, which can further impair microvascular perfusion, reduce the efficiency of oxygen delivery, and disrupt microcirculation, which may potentially contribute to tissue hypoxia, disease-related fatigue, and organ damage [33,34]. The process is further exacerbated by the fact that cyclophosphamide treatment damages the progenitor stem cells in the bone marrow where RBCs are formed and results in reduced RBCs production.

Three or more treatments cause significant changes in the structure of red blood cells, resulting in a less flexible structure. This may underline the hematological and cardiomyopathic side effects of RBCs, as well as oxidative damage resulting from an increase in free radicals and a decrease in antioxidants. Cyclophosphamide and its toxic metabolites (e.g., acrolein) induce oxidative stress, leading to lipid peroxidation of the RBCs membranes, leading to the lysis and extravasation of red blood cells, which when combined cause anemia [35,36]. Furthermore, DSC analysis and curve deconvolution reveal important qualitative findings regarding the manifestations of drug side effects on red blood cells, which routine laboratory tests are unable to detect.

The significance of this lies in the fact that oxidative damage, which is one of the origins of neoplastic processes that can be further exacerbated by anticancer therapies, plays an important role in altering RBCs adhesion. Reactive oxygen species (ROS) derived from leukemic metabolism and drug-induced stress can oxidize membrane lipids and cytoskeletal proteins, leading to lipid peroxidation, spectrin cross-linking, and clustering of membrane proteins. These oxidative modifications impair membrane flexibility, expose adhesion sites and phosphatidylserine residues, and enhance cell–cell and cell–matrix interactions [30,32,37,38,39]. Therefore, not only the disease itself, but also its treatment results in cellular disorganization of RBCs, which is unfortunately a well-known side effect associated with cytostatic therapy.

Cilostazol was developed in the 1980s by Takao Nishi’s laboratory at Otsuka and was first approved in Japan in 1988 in the treatment of peripheral arterial disease. In 1999, it received FDA approval in the United States and is marketed under the brand name Pletal. As a quinolinone derivative, it is a phosphodiesterase type 3 (PDE3) inhibitor that inhibits cellular breakdown of the second messenger cAMP. It works by inhibiting platelet aggregation and acting as a vasodilator to improve blood flow, thus being applicable for the symptomatic relief of intermittent claudication in patients afflicted with peripheral limb ischemia [40].

According to evidence-based medicine, cilostazol has a Class IA recommendation for the treatment of intermittent claudication. This is confirmed by feedback from patients using the medication: an improvement in walking distance becomes noticeable following 2–4 weeks, and the optimal effect is achieved after approximately 12 weeks. This corresponds to the observation period we recently used. Moreover, several clinical studies have demonstrated that 3-month cilostazol treatment improved quality of life and lower limb functional capacity among patients with intermittent claudication, regardless of age or sex. Meanwhile, its well-known side effects include headache and diarrhea [41,42,43].

In the second part of our study, a method for the thermal analysis and deconvolution of RBCs in patients with lower limb ischemia during a three-month cilostazol treatment was developed. The control DSC curve showed 5–6 distinct thermal domains, and in contrast to other drug treatments, this remained stable throughout the entire study period (except for the female group prior to initiating treatment). The similar structural relaxation of animal RBCs (decreased denaturation temperatures and calorimetric enthalpy as a function of treatment) induced by the anticancer drug cyclophosphamide was not observed in human red blood cells following cilostazol administration. The circulatory effects of cilostazol are currently understood to involve not only platelets but also red blood cell deformability, increasing their ability to deform and pass through capillaries, which helps to improve microvascular blood flow and reduce the symptoms of peripheral arterial disease. It exerts its effects by inhibiting PDE3, increasing cAMP, and probably normalizing red blood cell 2,3-diphosphoglycerate, leading to improved tissue oxygenation [44,45]. Additionally, there was a difference when contrasting sexes. In the male group, red blood cells showed greater thermal stability in calorimetric enthalpy than when compared with female patients. This difference may indicate different protein rearrangement patterns between the sexes, which could potentially affect red blood cell deformability, blood viscosity, and oxygen delivery efficiency, all of which are critical for efficient microcirculation in peripheral lower limb ischemia [46,47]. This may be supported by our observation [48] in which the main denaturation temperature range (the temperature difference between the native and denatured states) of cilostazol-treated patients increased with treatment time in plasma, suggesting a looser cooperation between plasma proteins, resulting in a decrease in viscosity.

In summary, this Special Issue focuses on the significance of the structure and function of red blood cells and provides a comprehensive overview of their critical importance. Given the significance of this topic from numerous perspectives, we felt it was important to compile our previous individual studies by presenting these findings into a single article. When placed side by side, the results clearly highlight not only how important it is to understand the effects of various drugs on target organs, but also the need to recognize how many other cells are affected by this process, including red blood cells. We consider it important to present the changes obtained using biophysical methods, thermal analysis, and DSC curve deconvolution, as these are procedures that many biologists, pharmacologists, and oncologists either do not know or do not use. Overall, these complementary biophysical approaches, including the deconvolution of DSC curves, highlight the importance of various drug treatments in elucidating changes in red blood cell membranes and hemoglobin abnormalities. Recent deconvolution analyses of curves have partially revealed the biological variations underlying the data; however, it needs more investigation.

## 5. Conclusions

Various drug treatments—in addition to their effects on target cells—affect RBCs, a fact that is often overlooked. The anticancer drug cyclophosphamide increased RBCs rigidity, thereby affecting their role in blood flow and their oxygen-carrying capacity, leading to anemia and cardiac complications. In contrast, cilostazol improves RBCs deformability and viscoelastic parameters of plasma proteins, thereby significantly improving patients’ quality of life and alleviating claudication symptoms in cases of lower extremity peripheral arterial disease. We endeavored to support these observations by DSC measurements and deconvolution of RBCs’ DSC scans.

## Figures and Tables

**Table 1:** The protocol of cyclophosphamide treatment of experimental animals.

Treatment	Time/Day															
	1	2	3	4	5	6	7	8	9	10	11	12	13	14	15	Ʃ
control	E															5 pcs
1 inj.	X	E														5 pcs
1 inj.	X				E											5 pcs
1 inj.	X						E									5 pcs
2 inj.	X	X	E													5 pcs
2 inj.	X	X				E										5 pcs
2 inj.	X	X						E								5 pcs
2 inj.	X				X				E							5 pcs
3 inj.	X	X						X				E				5 pcs
4 inj.	X	X						X				XE				5 pcs
5 inj.	X	X						X				X			XE	5 pcs
6 inj.	X	X						X				X	X	X	E	5 pcs

The symbols stand for: X = number of injections; E = exit + sampling.

**Table 2:** The characteristic average thermal parameters of the denaturation over the time of treated guinea pig RBCs samples.

Treatment	Thermal Parameters of Red Blood Cells			
	*T_m_*_1_/°C	*T_m_*_2_/°C	*T_m_*_3_/°C	Δ*H_c_*/Jg^−1^
**control**	68.9 ± 0.3	78.2 ± 0.4	84.4 ± 0.3	4.72 ± 0.26
**H** contribution	30.7%	19.9%	48.3%	98.3%
**one injection**				
**1i**-5d-E	68.4 ± 0.4	75.9 ± 0.4	84.0 ± 0.3	5.46 ± 0.26
**H** contribution	38.9%	18.6%	45.7%	103.2%
**three injections**				
**2i**-5d-**1i**-3d-E	67.4 ± 0.3	73.3 ± 0.3	-	3.97 ± 0.19
**H** contribution	62.4%	28.8%		91.2%
**five injections**				
**2i**-5d-**1i**-3d-**2i**-E	66.7 ± 0.3	71.8 ± 0.3	-	3.41 ± 0.18
**H** contribution	14.2%	93.5%		107.7%

The parameters present the following: **T_m_**: melting temperature; **ΔH_c_**: total calorimetric enthalpy of those thermal units which gave the largest contribution to the total calorimetric enthalpy. **i** represents the number of cyclophosphamide injections, **d** represents the untreated period in days, and **E** is the exit, while **H’s** contribution represents the enthalpy contribution of the thermal unit associated with a given denaturation temperature, expressed as a percentage. Each group contains minimum of five samples. Data rounded to one decimal place for temperature and two decimal places for calorimetric enthalpy. The unpaired, independent post hoc two-sample *t* test is based on the data in Table 1 in case of cyclophosphamide-treated samples (**i** stands for injection, d is the pause in days, and E is exit) exhibited among all parameters (which are the denaturation temperatures (except of T_m3_) and calorimetric enthalpy) significant differences between control and treated samples as well as those among the treated samples (at *p* < 0.006, control is green,  red represents an increasing and blue represents a decreasing trend). During chemotherapy, significant structural changes occur not only in the attacked tumor organ, but also in various components of the blood which carries the drug. We have not found direct experimental data regarding the binding of cyclophosphamide to the RBCs; however, thermal analysis interprets the increase in the denaturation temperature of a given thermal domain as a stiffening of its structure.

**Table 3:** The characteristic average thermal parameters of the denaturation over the span of time representing cilostazol treatment among female patients.

Treatment	Thermal Parameters of Female RBCs			
	*T_m_*_1_/°C	*T_m_*_2_/°C	*T_m_*_3_/°C	Δ*H_c_/*Jg^−1^
**healthy control**	68.7 ± 0.3	75.6 ± 0.4	81.8 ± 0.3	4.3 ± 0.20
H contribution	72.0%	14.1%	12.3%	98.4%
**0th week**	70.0 ± 0.4	78.0 ± 0.4	-	3.8 ± 0.16
H contribution	79.4%	19%		98.4%
**2nd week**	69.6 ± 0.3	78.0 ± 0.3	83.7 ± 0.4	4.1 ± 0.20
H contribution	53.9%	20.9%	23.4%	95.2%
**1 month**	70.1 ± 0.3	77.9 ± 0.3	83.5 ± 0.4	4.3 ± 0.20
H contribution	67.9%	18.8%	12.5%	94.2%
**2 months**	70 ± 0.3	78.5 ± 0.3	83.0 ± 0.4	4.9 ± 0.20
H contribution	66.8%	17.6%	11%	95.2%
**3 months**	70.2 ± 0.3	79.0 ± 0.3	84.0 ± 0.4	3.97 ± 0.19
H contribution	66.3%	21.9%	7.8%	96.0%

The parameters represent the following: ***T_m_***: melting temperature; **Δ*H_c_***: total calorimetric enthalpy of those thermal units which contributed to the largest contribution to the total calorimetric enthalpy. H contribution represents the enthalpy contribution of the thermal unit associated with a given denaturation temperature, expressed as a percentage. Each group contains minimum of five samples. Data rounded to one decimal place for temperature and two decimal places for calorimetric enthalpy. Control is green, unchanged contribution is black, significantly increased is red, and decreased is blue; compared with control.

**Table 4:** The characteristic average thermal parameters of the denaturation over the time of the cilostazol treatment among male patients.

Treatment	Thermal Parameters of Male RBCs			
	*T_m_*_1_/°C	*T_m_*_2_/°C	*T_m_*_3_/°C	Δ*H_c_/*Jg^−1^
**healthy control**	69.0 ± 0.3	76.7 ± 0.4	-	3.9 ± 0.20
H contribution	84.8%	11.2%		96.0%
**0th week**	70.0 ± 0.4	78.4 ± 0.4	-	4.2 ± 0.20
H contribution	75.6%	19.5%		95.1%
**2nd week**	70.0 ± 0.3	79.0 ± 0.3	84.0 ± 0.4	5.2 ± 0.20
H contribution	65.0%	26.8%	5.5%	97.3%
**1 month**	70.2 ± 0.3	78.6 ± 0.3	-	4.9 ± 0.20
H contribution	68.4%	25.6%		94.2%
**2 months**	70.0 ± 0.3	78.0 ± 0.3	83.0 ± 0.4	5.0 ± 0.20
H contribution	68.3%	28.6%	4.8%	101.7%
**3 months**	70.1 ± 0.3	78.5 ± 0.3	-	4.6 ± 0.18
H contribution	64.8%	28.6%		93.4%

The parameters represent the following: ***T_m_***: melting temperature; **Δ*H_c_***: total calorimetric enthalpy of those thermal units which contributed the largest contribution to the total calorimetric enthalpy. H contribution represents the enthalpy contribution of the thermal unit associated with a given denaturation temperature, expressed as a percentage. Each group contains a minimum of five samples. Data rounded to one decimal place for temperature and two decimal places for calorimetric enthalpy. Control is green, unchanged contribution is black, significantly increased is red, and decreased is blue, compared with the control.

## Data Availability

The study reported all data.

## Abbreviations

The following abbreviations are used in this manuscript:

cAMP	Cyclic adenosine monophosphate
DSC	Differential scanning calorimetry
FDA	Food and Drug Administration
H	Enthalpy contribution of the thermal unit
*ΔH_c_*	Total calorimetric enthalpy
Hb	Hemoglobin
PAM	Phosphoramide mustard
PDE3	Phosphodiesterase type 3
RBCs	Red blood cells
ROS	Reactive oxygen species
*T_m_*	Melting temperature
